# Prognostic Value of Genes and Immune Infiltration in Prostate Tumor Microenvironment

**DOI:** 10.3389/fonc.2020.584055

**Published:** 2020-10-30

**Authors:** Wenguo Sun, Hailin Shi, Zhen Yuan, Li Xia, Xuebao Xiang, Xiangfeng Quan, Wenjie Shi, Leiming Jiang

**Affiliations:** ^1^ Department of Urology, Affiliated Hospital of Guilin Medical University, Guilin, China; ^2^ Department of Urology, Fuyang People’s Hospital, Fuyang, China; ^3^ Department of Dermatology, Guilin People’s Hospital, Guilin, China; ^4^ Department of Gynecology, Pius Hospital of Oldenburg, Oldenburg, Germany

**Keywords:** prostate tumor, prognostic genes, immune infiltrate, tumor microenvironment, CXCR4, GPR183

## Abstract

**Background:**

Prostate cancer (PCa) is one of the most common cancers and the fifth leading cause of cancer-related death in men. Immune responses in the tumor microenvironment are hypothesized to be related to the prognosis of PCa patients; however, no studies are available to confirm the same. In this study, we aimed to explore the potential link between these two factors and identify new biomarkers to estimate the survival rate of PCa patients.

**Methods:**

A total of 490 cases were obtained from The Cancer Genome Atlas (TCGA) database. The gene expression data were analyzed by the ESTIMATE algorithm to evaluate the immune and stromal scores. The survival rate was calculated according to the case-specific clinical data. Enrichment analysis was performed to discover the main biological processes and signaling pathways of immune responses. We further identified and analyzed hub genes in the protein-protein interaction (PPI) network and evaluated their prognostic values.

**Results:**

Immune score significantly correlated with immune cell infiltration and overall survival of PCa patients. The genes CXCR4 and GPR183, identified as hub genes in the PPI network, correlated with immune cell infiltration and prognosis of PCa patients.

**Conclusion:**

CXCR4 and GPR183 participate in immune cell infiltration and function in PCa patients. The immune score, as well as the expression of CXCR4 and GPR183 in prostate cancer tissues, could be potential indexes for the prognosis of prostate cancer.

## Introduction

Prostate cancer (PCa) is one of the most common cancers of men and the fifth leading cause of cancer-related morbidity in men worldwide (1). PCa patients during the early stages are prescribed with radical prostatectomy and radiotherapy, which have a high rate of complete cure (2). For metastatic PCa patients, surgery or androgen deprivation is advised. However, androgen deprivation therapy or androgen receptor-targeted therapy can also induce tumor resistance (3). Therefore, new and promising therapeutic strategies for PCa are required. Anti-tumor immunotherapy with CTLA-4-targeted monoclonal antibodies is now under clinical investigation (4, 5). However, the precise relationship between the prognosis of PCa and the immune responses is still unknown.

Currently, prostate-specific antigen (PSA) values, histopathological scores such as Gleason score, are used as clinical parameters to diagnose PCa and assess the risk stratification (6). However, PSA is not a tumor-specific marker and the specificity of PSA is only 12.8% (7). This led to the introduction of prostate health index (PHI) to elevate the diagnostic capacity of PSA (8). The Oncotype DX Genomic Prostate Score (GPS) assay was used in clinical trials to make a risk assessment and predict the time of recurrence (9, 10). But this multi-gene assay may be unable to select the active surveillance candidates (11). Identification of specific markers with significant potential for the prognosis of PCa in terms of immune response is necessary.

The tumor microenvironment (TME) of the PCa patients is associated with inflammation (12). An increasing number of studies have reported that infiltrated immune cells play a pro-tumorigenic or anti-tumorigenic role in the TME of PCa patients (13–17). The Cancer Genome Atlas (TCGA) database has provided a series of global gene expression profiles and clinical data of the patients worldwide (18). Yoshihara et al. developed the ESTIMATE algorithm to assess the expression levels of specific molecular entities in stromal and immune cells of the TME (19), so that the non-tumor cell infiltrations in the TME can be predicted. Shah et al. used the ESTIMATE algorithm to evaluate the stromal score of prostate cancer; however, the investigators did not analyze the TCGA database or compare the survival rate in different groups based on estimated score (20).

In this study, we explored the prostate cancer data from TCGA databank, calculated the immune and stromal scores of every sample by ESTIMATE algorithm, and estimated their potential values of prognosis for PCa patients. To identify some specific genes to forecast the overall survival rate of the PCa patients, we performed enrichment analysis, and interaction analysis and identified two hub genes. These hub genes were found to be highly correlated with the prognosis of PCa and associated with tumor purity and immune infiltration. We hypothesized that these two genes can be the potential biomarkers for prognosis of PCa and guide the selection of immunotherapy strategy for the PCa patients. Further, these genes may also play a significant role in the underlying molecular mechanisms of PCa.

## Materials and Methods

### Raw Data From TCGA Database

We searched the TCGA database (https://portal.gdc.cancer.gov) and downloaded prostate cancer datasets after restricting the disease types to adenomas and adenocarcinomas. A total of 490 cases were included in this dataset that contained the gene expression files and clinical data of every patient. These gene expression files were processed by the ESTIMATE algorithm, (19) and the immune, stromal, and ESTIMATE scores of each sample were calculated. Since we only use the data from public online database in this study, so the ethic approval was not required.

### Survival Rate Analysis

Included participants were divided into two groups according to their immune, stromal, and ESTIMATE scores, and the expression levels of the candidate genes (separated by median values). We used Kaplan-Meier correlation analysis (95% CI) to evaluate the association between the overall survival rate and the immune, stromal, and ESTIMATE scores, and the identified genes expression levels. A log-rank test was used to check the significance of the relationships. The analyses were carried out using *R* software (version 3.6.1), and *P*<0.05 was considered statistically significant.

### Correlation Analysis

Included participants were divided into sub-groups to evaluate the relationships between different tumor stages including T1-T4, N0-N1, and M0-M1 stages and the estimated immune, stromal, and ESTIMATE scores. Wilcoxon signed-rank test was used to evaluate the relationship between the two groups; the Kruskal-Wallis test was used to evaluate the relationships among three or more groups. The analyses were conducted using *R* (version 3.6.1), and *P*<0.05 was considered statistically significant.

### Identification and Cluster Analysis of Differentially Expressed Genes

Gene expression files were processed by *limma* package (21) of *R* and the differentially expressed genes (DEGs) in immune-score-high and stromal-score-high groups were identified. The cut-off values were set as |fold change|>2 and adjust. *P*<0.05. Cluster analysis and heatmaps were generated by *pheatmap* package (22) of *R*, and the upregulated genes in both the high-rank groups were calculated by the *VennDiagram* package (23). Further, we analysed the immune cell specific markers such as CD14 (monocytes) and CD3 (T cells) between the two groups.

### Enrichment Analysis

Gene Ontology (GO) enrichment analysis and Kyoto Encyclopedia of Gene and Genomes (KEGG) pathway enrichment analysis of the upregulated genes were performed by *R* Packages *clusterProfiler* (24), *enrichplot*, and *ggplot2* (25), and the significantly activated biological processes, molecular functions, cellular components, and signaling pathways were explored.

### Protein-Protein Interaction (PPI) Network and Hub Genes Calculation

*STRING* tool (https://string-db.org/) was used to construct the Protein-Protein Interaction (PPI) network of the identified genes (CI=0.90). The network was reconstructed using *CytoHubba*, a plug-in of *Cytoscape* software (version 3.5). Using this software, we calculated the connection degree of every node in the network to identify the top 30 hub genes.

### Specific Gene Expression Comparison and Immune Infiltration Analysis

*TIMER* tool (https://cistrome.shinyapps.io/timer/) was used to compare the specific gene expression levels between the tumor tissues and normal control tissues of different carcinomas. The correlation between specific gene expression and infiltration of different immune cells in PCa tissues was assessed by purity-corrected partial Spearman’s correlation analysis. Also, the relationship between the two particular genes was calculated by Spearman’s correlation analysis. *P*<0.05 was considered statistically significant.

### Gene Set Enrichment Analysis (GSEA)

The gene expression files of the 490 cases downloaded from TCGA was separated into CXCR4 (or GPR183) high-express group and low-express group. Gene set enrichment analysis for KEGG pathway was conducted by using *GSEA* tool (https://www.gsea-msigdb.org/gsea/index.jsp). *P*<0.05 was considered as statistically significant.

### GEO Datasets Analysis

We searched GEO database and downloaded the human PCa-related datasets. The transcriptome of CXCR4 and GPR183 were compared between tumor and paired normal tissues (GSE69223) (26), recurrent patients, and non-recurrent patients after surgical operation (GSE25163) (27), as well as castration-resistent patients and good prognosis patients (GSE37199) (28). *P*<0.05 was considered as statistically significant.

## Results

### Correlation Between Immune Score and Prognosis of Prostate Cancer Patients

Transcriptional expression files and clinical data of 490 prostate cancer (adenomas and adenocarcinomas) patients were downloaded from the TCGA databank. Out of these patients, one was American Indian (0.20%), 12 were Asian (2.45%), 58 were African American (11.84%), 407 patients were white (83.1%), and the race of rest of the 12 patients (2.41%) was unknown. All the patients whose data is included in this study were diagnosed with adenomas or adenocarcinomas.

Using the ESTIMATE algorithm, we evaluated the stromal, immune, and ESTIMATE scores of every sample. The stromal score of PCa patients varied from -1,925.26 to 1771.63, and the immune score varied from -1,339.23 to 1,646.97. The total ESTIMATE score, which is the combination of stromal score and immune score, ranged from -3,264.49 to 3,418.60.

**Figure 1:**
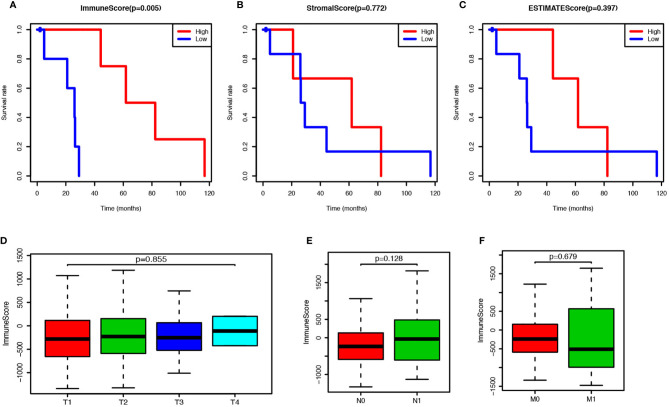
Prostate cancer (PCa) patients with higher immune score have better prognosis. **(A)** PCa patients were divided into two groups according to the immune scores, and the survival rate of these two groups was shown as Kaplan-Meier survival curve, which indicated the PCa patients with higher immune score have better prognosis (*P* = 0.005). **(B)** PCa patients were divided into two groups according to the stromal scores, and their survival rates did not show any significant difference (*P* = 0.772). **(C)** PCa patients were divided into two groups according to the ESTIMATE scores, and there was no significant difference on survival rates between these two groups (*P* = 0.397). **(D)** Immune scores of PCs patients in each tumor stage were shown by box-plot, but no significant association between the immune score and tumor stage was found (*P* = 0.855). **(E)** Immune scores of PCs patients with or without lymph node metastasis were shown by box-plot, but there was no significant difference between the two groups (*P* = 0.128). **(F)** Immune scores of PCs patients with or without metastasis were shown by box-plot, and no significant difference was shown between the two groups (*P* = 0.128).

To estimate the prospective relationship between the overall survival of PCa patients and their stromal, immune, and ESTIMATE scores, we separated the selected cases into a high-rank group and low-rank group based on these scores and compared the survival rate in the two groups. Intriguingly, the Kaplan-Meier survival curves revealed that PCa patients with higher immune scores live longer than those with lower scores (**Figure 1A**). Moreover, no significant differences were observed in the prognosis between stromal-scores-high and stromal-scores-low groups and between ESTIMATE-scores-high and ESTIMATE-scores-low groups (**Figures 1B–C**). So, we further compared the immune scores among the PCa patients under different stages, but no significant differences were observed in the immune scores among the different T stages, N stages, and M stages (**Figures 1D–F**). Further, we found that the immune score is significantly correlated with the overall survival of PCa patients, and it could be used as a potential index to forecast the prognosis of PCa.

### Immune-Related Genes Were Highly Expressed in High-Rank Groups of Immune Scores and Stromal Scores

**Figure 2:**
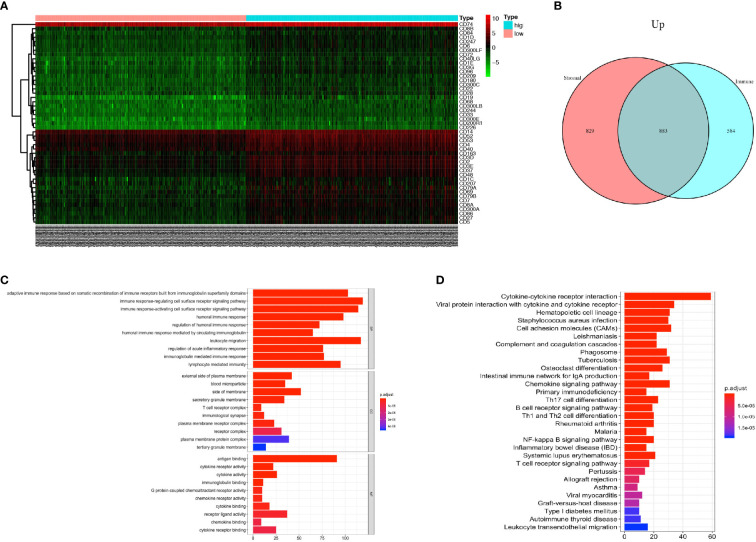
PCa patients with higher immune score have more immune cell infiltration and more activated immune responses. **(A)** Heatmap of the CD markers gene expression levels between the immune score high group and low group (*P* < 0.05, Fold Change>2). **(B)** The number of commonly upregulated genes in immune score high group and stromal score high group shown by Venn diagram. **(C)** Top 10 GO terms, including BP, CC, and MF, respectively, enriched according to the commonly upregulated genes (*P* < 0.05). **(D)** Top 20 KEGG terms enriched according to the commonly upregulated genes (*P* < 0.05).

DEGs analysis was performed using *R* software to compare the DEGs between the high rank and the low-rank groups of immune score and stromal score. A total of 1467 genes were found upregulated and 9 genes were found downregulated in the immune-scores-high group (**Supplement Figure 1**); 1,712 genes were found upregulated and 14 genes were found down-regulated in the stromal-scores-high group (**Supplement Figure 2**). The expressions of immune cell CD markers were also estimated. Expression levels of cell-specific markers such as CD14 (monocytes), CD3 (T cells), CD4, CD8, CD19 (B cells), and CD163 (macrophages); co-stimulatory factors such as CD28 and CD40; cell activation markers such as CD48 and CD79, were found to be higher in the immune-scores-high group as compared with an immune-scores-low group (**Figure 2A**). Moreover, 883 genes were found to be commonly upregulated in the immune-scores-high group and the stromal-scores-high group (**Figure 2B**).

Next, we performed GO and KEGG enrichment analyses on these 883 commonly upregulated genes to identify their main functions. The top 10 of GO items including BP, CC, and MF items are listed in **Figure 2C**. The most significantly enriched GO items were adaptive immune response (GO: BP), plasma membrane (GO: CC) and antigen binding (GO: MF). Besides, these commonly upregulated genes were highly enriched in cytokine and cytokine receptor interaction, followed by items such as Th17 cell differentiation, Th1, and Th2 cell differentiation, and so forth (**Figure 2D**).

In conclusion, the PCa tumors with higher immune scores have more immune cell infiltration with monocytes, T-cells, B-cells, and macrophages. Also, the commonly upregulated genes pointed toward activated immune responses in the TME.

### CXCR4, a Hub Gene in the PPI Network, Correlated With PCa Prognosis

**Figure 3:**
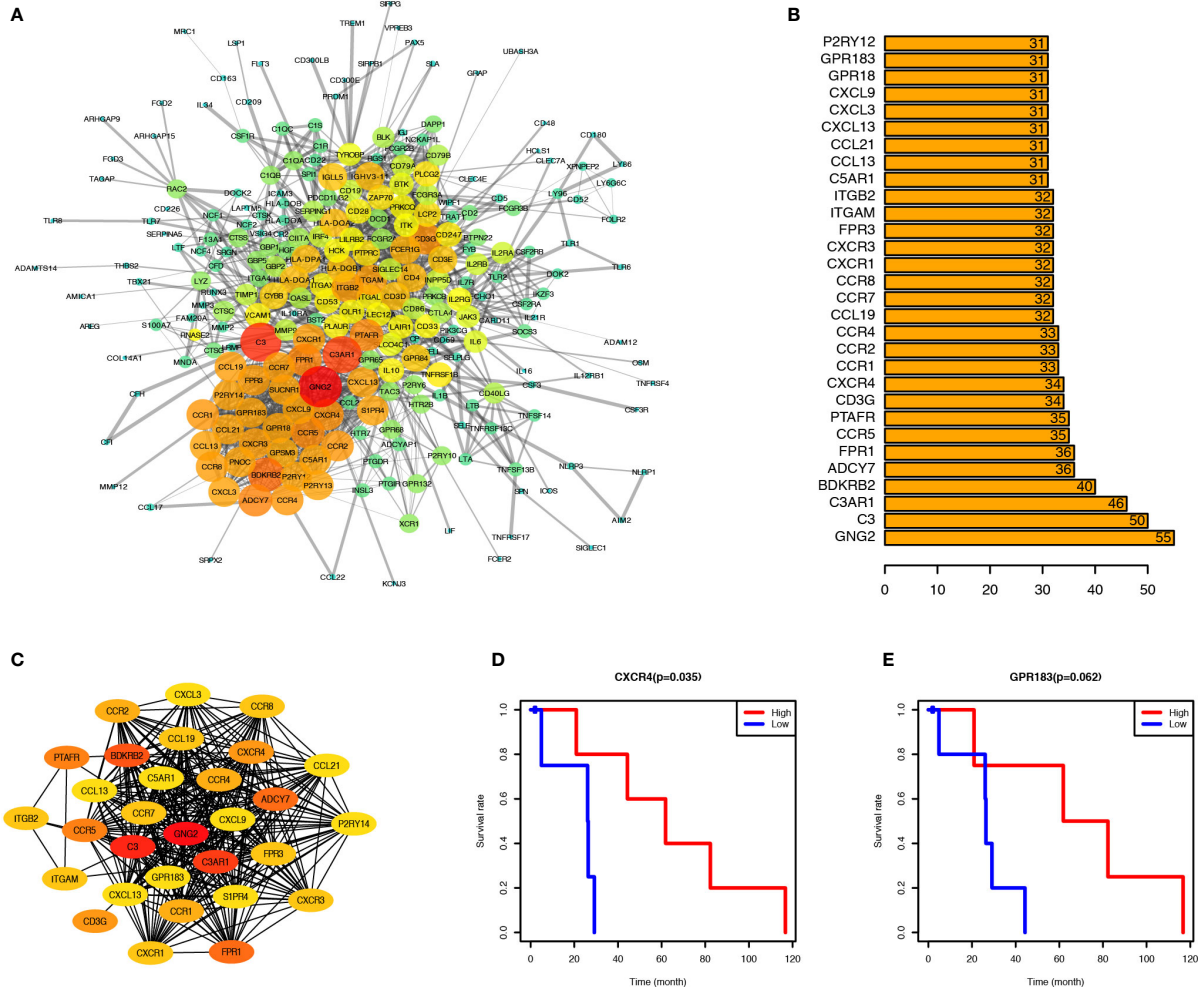
CXCR4 and GPR183 were hub genes from the Interaction Network of upregulated genes, and associated with PCa prognosis. **(A)** Protein-Protein Interaction Network (CI=0.90) based on the upregulated genes, and the size and color of node indicate the connection degree. **(B)** Top 30 hub genes calculated by the connection degree of each node. **(C)** Interaction Network of top 30 hub genes. **(D)** PCa patients were divided into two groups according to the CXCR4 expression level, and the survival rate of these two groups indicated the PCa patients with higher CXCR4 expression level have better prognosis (*P* = 0.035). **(E)** Similarly, the PCa patients with higher GPR183 expression level have better prognosis, although it was not significant (*P* = 0.062).

PPI network was created through the STRING tool to analyze the connections of identified genes. GNG2, C3AR1, and C3 were located at the center of the network. Most chemokine and chemokine receptors such as CXCL3, CCR4, CXCR4, and CXCR3 were found to be tightly connected and located mainly at the lower-left region (**Figure 3A**); cytokine and cytokine receptors such as IL6, IL10, IL2RA, and IL2RB, co-stimulatory factors such as CD28, and CTLA4, and other important factors including IRF4, JAK3, and BTK were located at the upper-right region of the main network. To identify the critical genes in this network, we calculated the connection degree of each node in the network and identified the top 30 hub genes (**Figure 3B**). GNG2, with 55 connections with other nodes, was the most highly connected one, followed by C3, C3AR1, BDKRB2, ADCY7, FPR1, CCR5, PTAFR, FPR3, and CXCR4. We built a new network based on these 30 hub genes using CytoHubba plug-in of Cytoscape (version 3.5) (**Figure 3C**).

To identify the potential prognostic markers of PCa, we analyzed the relationship between the expression levels of these 30 hub genes and the prognosis of PCa (**Figures 3D, E**, and **Supplement Figure 3**). CXCR4 was found to be correlated with PCa prognosis (**Figure 3D**), and PCa patients with elevated expression of CXCR4 in tumor tissues have a higher survival rate than those with relatively low expression of CXCR4. Besides, GPR183 was another critical gene, which tended to be upregulated in PCa patients with a better prognosis (**Figure 3E**), but the difference in the GPR183 expression levels between PCa and normal control patients was not statistically significant (*P*=0.062). However, there is no significant differences of CXCR4 and GPR183 expression levels among different tumor stage patients (**Supplement Figure 4**).

Therefore, CXCR4 and GPR183 can be potential candidate biomarkers to forecast the prognosis of PCa patients.

### CXCR4 and GPR183 Are Down-Regulated in PCa Tissue and Are Associated With Immune Cell Infiltration in PCa Tumors

**Figure 4:**
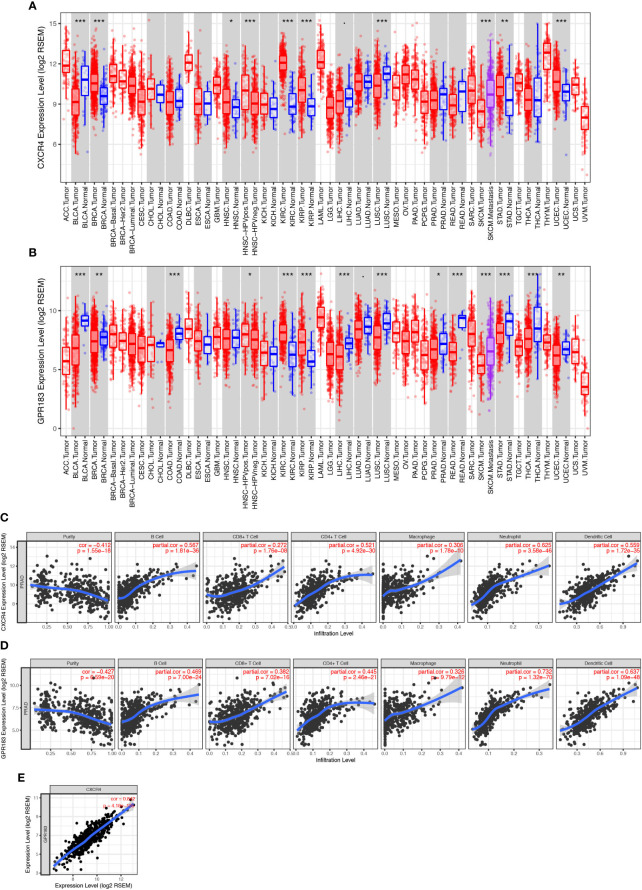
CXCR4 and GPR183 correlated with immune cell infiltration in PCa tissue. **(A)** CXCR4 expression levels in different kinds of tumors and their control tissues according to the TCGA data, and there was no significant difference between prostate cancer tissue and its control normal tissue. **(B)** GPR183 expression levels in different kinds of tumors and their control tissues according to the TCGA data, and GPR183 was significantly decreased in prostate cancer tissue (*P* < 0.05). **(C)** Correlation analysis on CXCR4 and the tumor purity (Correlation Index=-0.412, *P* < 0.001) and immune cell infiltration of prostate cancer. The higher expression level of CXCR4 in tumor tissue was accompanied by increased infiltrated B cells, CD8+ T cells, CD4+ T cells, macrophages, neutrophils, and dendritic cells (*P* < 0.001). **(D)** Correlation analysis on GPR183 and the tumor purity (Correlation Index=-0.427, *P* < 0.001) and immune cell infiltration of prostate cancer. The higher expression level of CXCR4 in tumor tissue was accompanied by increased infiltrated B cells, CD8+ T cells, CD4+ T cells, macrophages, neutrophils, and dendritic cells (*P* < 0.001). **(E)** The expression level of CXCR4 and GPR183 in prostate cancer tissue was highly correlated (Correlation Index=0.842, *P* < 0.001).

We used the TIMER tool to elucidate the role of CXCR4 and GPR183 in PCa tumors. We compared the expression levels of the different genes from the tumor and the normal tissues (control) using the information from the TCGA databank. The expression level of CXCR4 was found to be similar between PCa tumor and normal tissues (**Figure 4A**); however, GPR183 was down-regulated in the PCa tumor tissues (**Figure 4B**) as compared with the normal tissues. To confirm the different expression pattern, we searched the GEO database and reanalyzed the GSE69223 dataset (26), which compare the transcriptome of PCa tissue and paired normal tissues. Consequently, CXCR4 and GPR183 are both found to be downregulated in PCa tissues (**Supplement Figure 5**).

According to the dataset of GES25136 (27), we analyzed the expression of CXCR4 and GPR183 in tumor of recurrent PCa patients and non-recurrent PCa patients. No significant differences of CXCR4 and GPR183 expression pattern was found between these two groups (**Supplement Figure 6**).

Further, neither CXCR4 nor GPR183 show a significantly different expression level in the blood of castration-resistant PCa patients and good prognosis PCa patients, when we reanalyzed the data from GSE37199 (28) (**Supplement Figure 7**).

Next, partial Spearman’s correlation analysis was used to assess the relationship among the expression of gene CXCR4, gene GPR183, and the immune cell infiltration levels in PCa tumors. The CXCR4 expression level was significantly associated with purity (correlation=-0.412, *P*<0.001) and positively correlated with the infiltration of B cells, CD4+ T-cells, CD8+ T-cells, macrophages, neutrophils, and dendritic cells (**Figure 4C**). Besides, GPR183 expression level had a negative relationship with purity (correlation=-0.427, P<0.001); infiltration of B cells, CD4+ T-cells, CD8+ T-cells, macrophages, neutrophils, and dendritic cells increased with the increase in expression of GPR183 (**Figure 4D**). Upon comparing the expressions of both CXCR4 and GPR183 in PCa tumors, we observed a high correlation (**Figure 4E**).

In addition, we separated the transcriptome data of the 490 cases into CXCR4-high (bigger than the median of all cases) and –low (smaller than the median of all cases) groups and GPR183-high and –low groups, respectively, to do gene set enrichment analysis (GSEA). Intriguingly, GSEA for KEGG pathway indicated that the CXCR4-high group are mostly enriched in Cytokine-Cytokine Receptor Interaction (M9809), Nature Killer Cell Mediated Signaling (M5669), Toll-Like Receptor Signaling (M3261), T Cell Receptor Signaling (M9904), and so on (**Supplement Figure 8**). Similarly, GPR183-high group are mainly enriched in MAPK Signaling (M10972), Cytokine-Cytokine Receptor Interaction (M9809), Toll-Like Receptor Signaling (M3261), and Nature Killer Cell Mediated Signaling (M5669), etc. (**Supplement Figure 9**). Therefore, we considered that CXCR4 and GPR183 have a deep relationship with immune reaction and immune cell function in PCa tissue.

In conclusion, CXCR4 and GPR183 were down-regulated in PCa tumors, and both of these genes were associated with tumor purity and immune cell infiltration.

## Discussion

Prostate cancer is the second most common tumor among males worldwide (29). The incidence of PCa in the Chinese population is much lower than that of European and American countries, but the cases are substantially rising since the beginning of the 21^st^ century (30, 31). Since some immunotherapy-based therapies have started showing promise in PCa treatment (32, 33), the pro-tumorigenic or anti-tumorigenic role of infiltrated immune cells attracted more attention than ever before. However, there is a lack of valid prognostic biomarkers to evaluate the immune status of TME and predict the survival rate of PCa patients.

Using the TCGA databank, we analyzed the expression profiles of prostate cancer patients, explored the TME, and selected hub genes with significant prognostic value. Further, we assessed the immune, stromal, and ESTIMATE scores of the PCa patients using the ESTIMATE algorithm and divided them into two groups (high-rank group and low-rank group) according to the median values of the scores. We compared the survival rates of the high and low-rank groups and observed that the PCa patients with higher immune score live longer than those with a lower score, contrary to the results reported for the other tumors such as glioblastoma and breast cancer (34–36). These variations might be attributed to the type of immune cells in TME since the majority of the T-cells identified in glioblastoma and breast cancer tissues are regulatory T-cells or exhausted effect T-cells. Immune escape is one of the most critical reasons for tumorigenesis, and immune cell infiltration can support the chemotherapies (37). For instance, tumors with an increased number of CD8^+^ T-cells along with some Foxp3^+^ Treg cells infiltrated in the TME are more likely to respond to chemotherapies (38). Further, we compared the immune scores according to the tumor stages, but no significant differences were observed among the different T stages, N stages, and M stages. Since the development and transformation of tumor cells depend more on their characteristics rather than the immune cell infiltration and reactions, immune scores make less contribution in evaluating the tumor stages.

After comparing the expression profiles of the prostate tumor tissues, we found a total of 883 genes to be commonly upregulated in the stromal/immune score high-rank groups. A large number of immune-related CD markers were found to be highly expressed in the immune score high group. Therefore, we concluded that the monocytes, T-cells, B-cells, and macrophages may infiltrate the TME, consistent with a published study that used mass cytometry to identify the immune cells in the human prostate (39). As per the GO- and KEGG enrichment analysis, many of the upregulated genes were found to be involved in the immune responses such as adaptive immune response, immune-regulating cell surface receptor signaling pathway, and cytokine-cytokine receptor interaction. After selecting the 30 hub genes from the commonly upregulated genes by analyzing the PPI network, we performed survival rate analyses according to the expression levels of these 30 hub genes. CXCR4 was found to be correlated with PCa prognosis, and GPR183 was found to be upregulated in PCa patients with a better prognosis. Both TCGA dataset and GEO dataset confirmed the lower expression levels of CXCR4 and GPR183 in PCa tissue when compared with normal tissue. However, the mortality of PCa patients is very low, so that the number of samples in survival curve is a limitation in this research. We believe a follow-up study based on a large cohort of PCa patients is necessary to confirm the prognosic value of CXCR4 and GPR183.

CXCR4 is a chemokine receptor mainly expressed in most hematopoietic cells and is specific for stromal cell-derived factor-1 (SDF-1) (also known as CXCL12) (40, 41). SDF-1 activates cells through the receptors CXCR4 and CXCR7, and these two receptors are expressed in different tumors either individually or in combination (42). CXCR7 rather than CXCR4 is expressed on most of the human glioblastoma cells and small-cell lung cancer cells (43). CXCR4 is a critical receptor to modulate tumor-stromal interactions including cell invasion and migration, and therapeutic resistance (44), and blocking of CXCR4 by AMD3100 increases the anti-tumor effect of docetaxel in PCa patients with tumor metastasis (45). But, we should not ignore that the high expression of CXCR4 was only observed in bone metastasis lesions and that the difference between the total expression levels of CXCR4 in PCa tissues *in situ* and the normal control tissues was not statistically significant (**Figure 4A**). The CXCR4/CXCL12 axis plays a key role in immune surveillance of tissues (46), since CXCR4 is expressed in macrophages, monocytes, T lymphocytes, B lymphocytes, and neutrophils these cells can be recruited by the expression of CXCL12 by stromal cells of TME. Thus, while separating the PCa patients by the expression levels of CXCR4, we found that the patients with a higher level of CXCR4, which may recruit more immune cells, have a better prognosis. The CXCR4 expression level was found to be significantly associated with tumor purity and positively correlated with the infiltration of B-cells, CD4+ T-cells, CD8+ T-cells, macrophages, neutrophils, and dendritic cells (**Figure 4C**). However, the exact immune cell atlas in the TME of PCa patients with different CXCR4 expression profiles need to be further identified and compared to confirm the potential phenotypical and functional differences in the infiltrating immune cells.

GPR183 (or EBI2), upregulated in primary B lymphocytes after Epstein-Barr virus (EBV) infection, is predicted to encode a G-protein coupled receptor that is closely related to the thrombin receptor (47). GPR183 plays an important role in promoting B-cell localization to the outer follicle and mediating B-cell migration, together with CXCR5, to regulate the germinal center reactions (48, 49). Besides, GPR183 can promote follicular helper T (Tfh) cells differentiation through the positioning of the activated T cells at the follicle-T-zone interface and mediating the dendritic cells to induce T/B cell responses (50–52). However, no studies have reported any links between GPR183 and prostate cancer. This might be because the expression of GPR183 is mostly specific to the immune cells especially to the B cells, and most of the earlier research about PCa did not focus on the immune cells in TME. In the present study, GPR183 was found to be downregulated in PCa tissues as compared with normal control tissues and upregulated in both the high-rank groups (immune score- and stromal score high). Besides, GPR183 expression levels negatively correlated with purity and positively correlated with the infiltrated B-cells, CD4+ T-cells, CD8+ T-cells, macrophages, neutrophils, and dendritic cells (**Figure 4D**). These findings may indicate that the B cells in TME of the prostate tumors are more active in migration. The group with lower scores, as well as lower expression of GPR183, showed a significantly decreased survival rate that may be attributed to the higher level of migration. However, further studies are needed to elucidate the exact underlying mechanisms.

In conclusion, using the ESTIMATE algorithm, we estimated the immune and stromal scores of the TCGA PCa cohort and concluded that the patients with a higher immune score have a better survival rate. CXCR4 and GPR183 are the two hub genes with significant prognostic value for PCa patients, which may attribute to their contribution to the immune cell infiltration and immune reaction.

## Data Availability Statement

Publicly available datasets were analyzed in this study. This data can be found here: https://portal.gdc.cancer.gov.

## Author Contributions

Conceptualization: LJ. Data curation: WGS. Formal analysis: WGS and HS. Funding acquisition: LJ, WJS, and WGS. Investigation: HS and WGS. Methodology: HS, WGS, and ZY. Project administration: LJ and WJS. Software: ZY and XX. Supervision: LJ and WJS. Validation: LX, XQ, and WJS. Visualization: WGS, ZY, and XX. Writing—original draft: HS and WGS. Writing—review and editing: LJ and WJS. All authors contributed to the article and approved the submitted version.

## Conflict of Interest

The authors declare that the research was conducted in the absence of any commercial or financial relationships that could be construed as a potential conflict of interest.

## Supplementary Material

Supplementary Figure 1 Heatmap of differently expressed genes of immune score high group and low group.

Supplementary Figure 2 Heatmap of differently expressed genes of stromal score high group and low group.

Supplementary Figure 3 Survival rate of the top 30 hub genes’ high-express group and low-express group.

Supplementary Figure 4 Expression level of CXCR4 and GPR183 among different tumor status (T0, T1, T2, N0, N1 and M0, M1) of PCa patients.

Supplementary Figure 5 Expression level of CXCR4 and GPR183 between tumor and paired normal tissue of prostate from PCa patients. This data is re-analysied by us according to the GEO dataset GSE69223.

Supplementary Figure 6 Expression level of CXCR4 and GPR183 in PCa tissue between recurrent and non-recurrent patients. This data is re-analysied by us according to the GEO dataset GSE25136.

Supplementary Figure 7 Expression level of CXCR4 and GPR183 in blood between castration resistant patients and non-resistent patients. This data is re-analysied by us according to the GEO dataset GSE37199.

Supplementary Figure 8 Gene set enrichment analysis (GSEA) for the CXCR4 high-expressed group of PCa. Top10 enriched KEGG pathways are shown in this figure.

Supplementary Figure 9 Gene set enrichment analysis (GSEA) for the GPR183 high-expressed group of PCa. Top10 enriched KEGG pathways are shown in this figure.
