# Supplementary figures and images for: Prognostic Value of Genes and Immune Infiltration in Prostate Tumor Microenvironment
Source: Front Oncol. 2020 Oct 30;10:584055. doi: 10.3389/fonc.2020.584055 (PMC7662134; doi:10.3389/fonc.2020.584055)

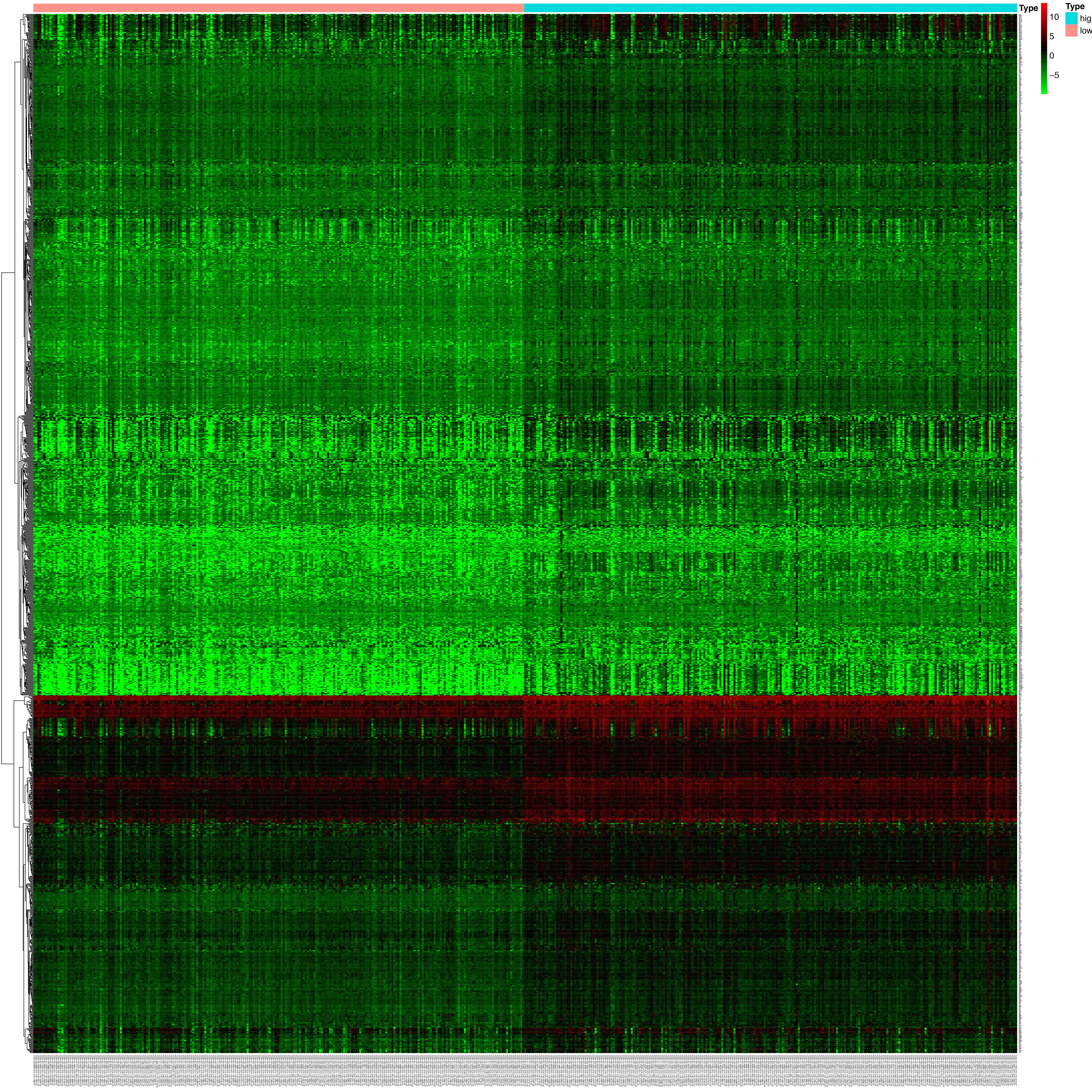

Supplement: Supplementary Figure 1 — Heatmap of differently expressed genes of immune score high group and low group. [file Image_1.jpg]

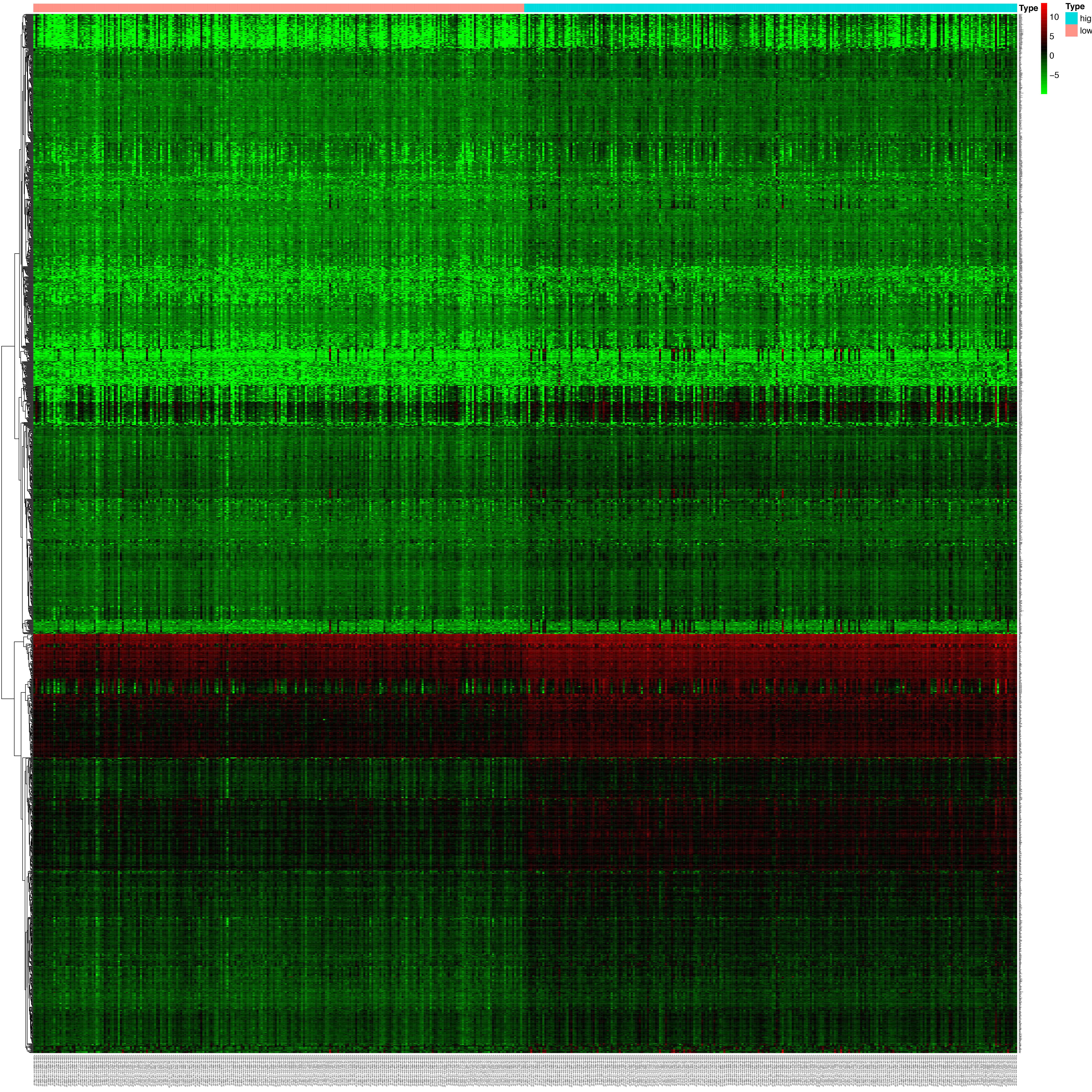

Supplement: Supplementary Figure 2 — Heatmap of differently expressed genes of stromal score high group and low group. [file Image_2.jpg]

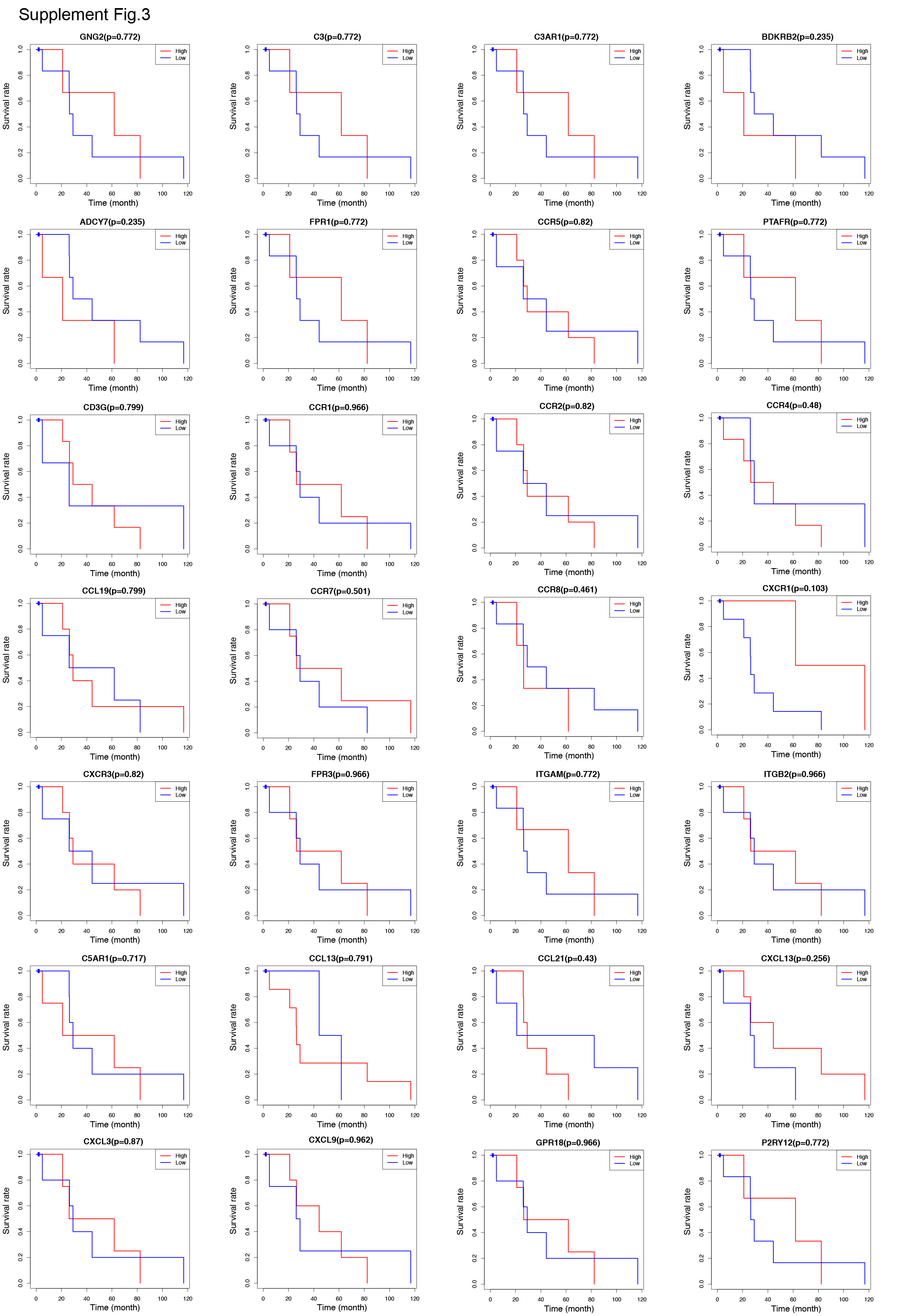

Supplement: Supplementary Figure 3 — Survival rate of the top 30 hub genes’ high-express group and low-express group. [file Image_3.jpg]

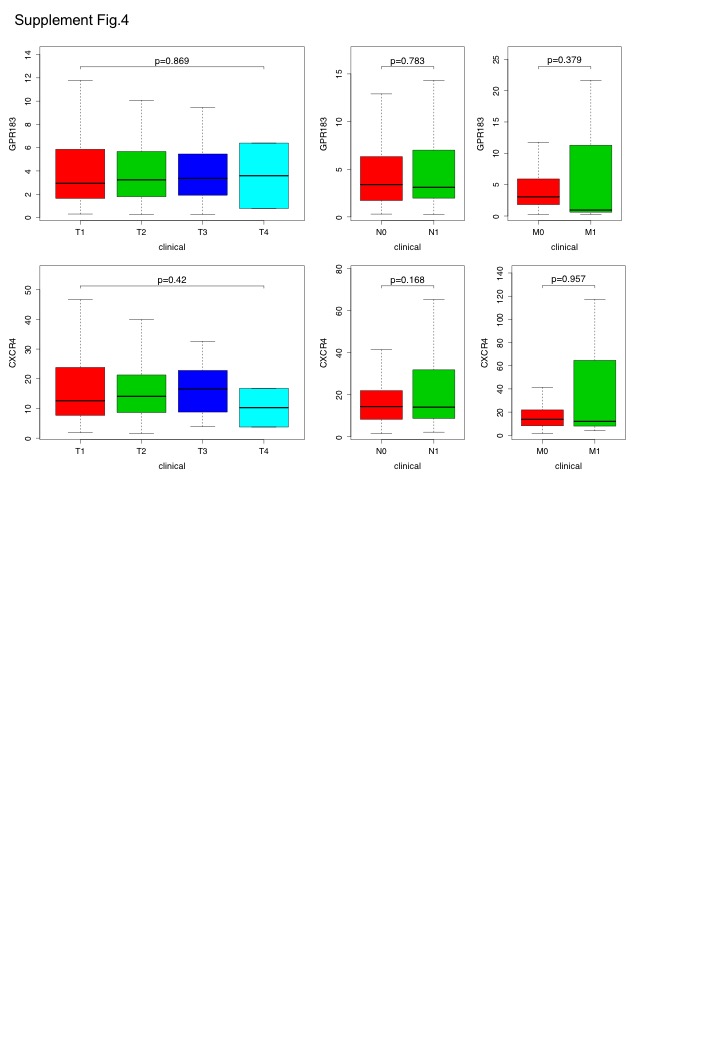

Supplement: Supplementary Figure 4 — Expression level of CXCR4 and GPR183 among different tumor status (T0, T1, T2, N0, N1 and M0, M1) of PCa patients. [file Image_4.jpg]

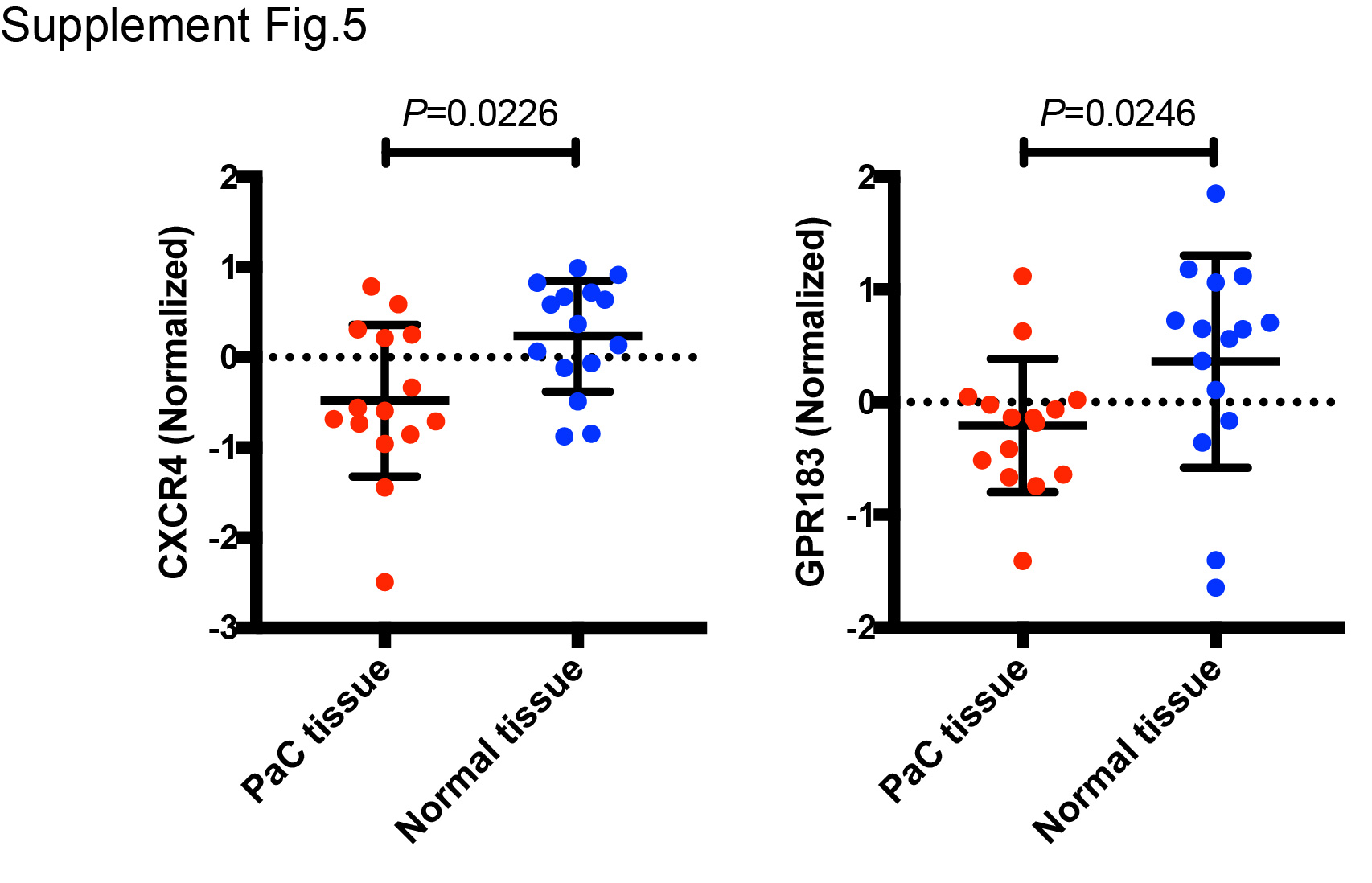

Supplement: Supplementary Figure 5 — Expression level of CXCR4 and GPR183 between tumor and paired normal tissue of prostate from PCa patients. This data is re-analysied by us according to the GEO dataset GSE69223. [file Image_5.jpg]

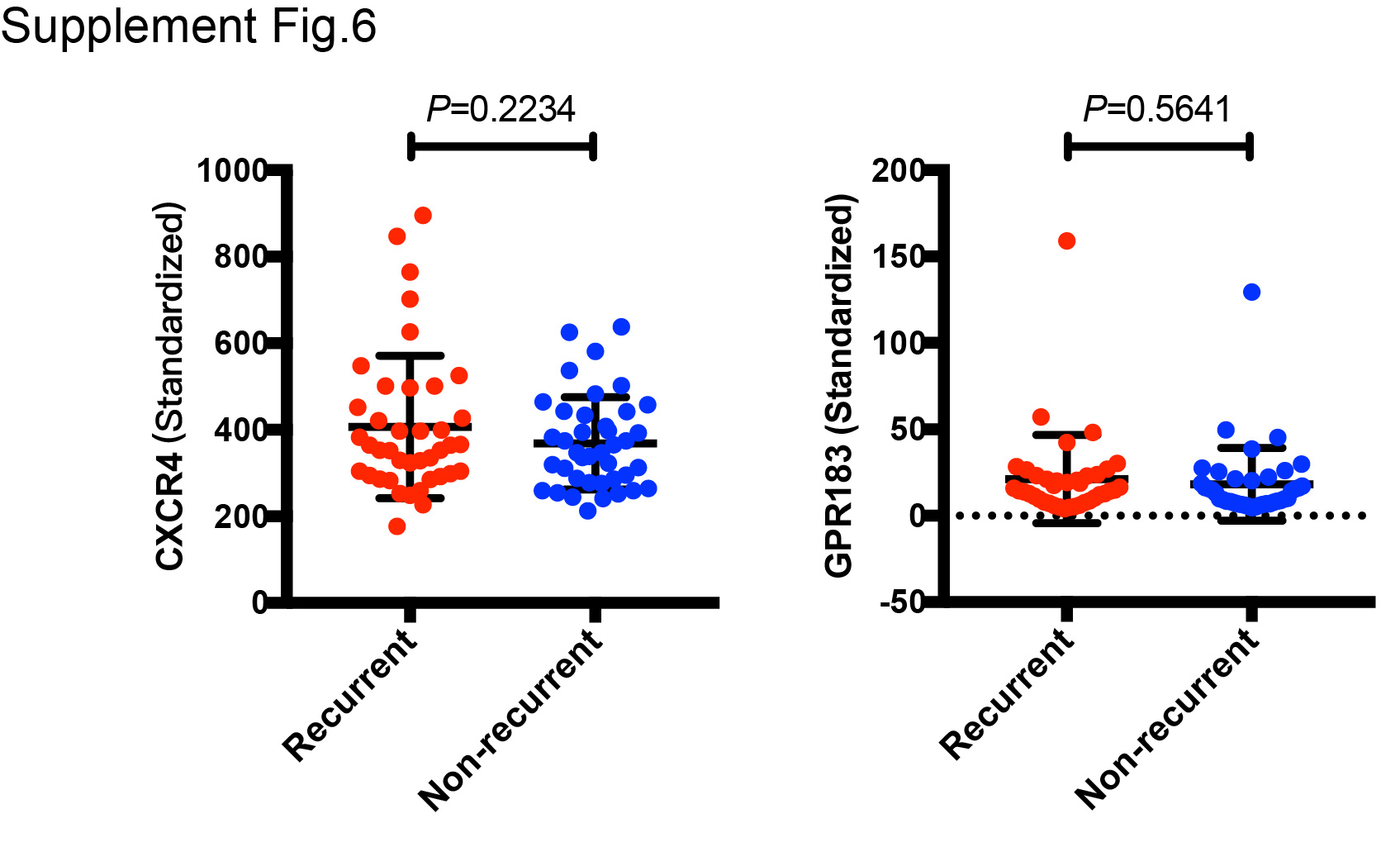

Supplement: Supplementary Figure 6 — Expression level of CXCR4 and GPR183 in PCa tissue between recurrent and non-recurrent patients. This data is re-analysied by us according to the GEO dataset GSE25136. [file Image_6.jpg]

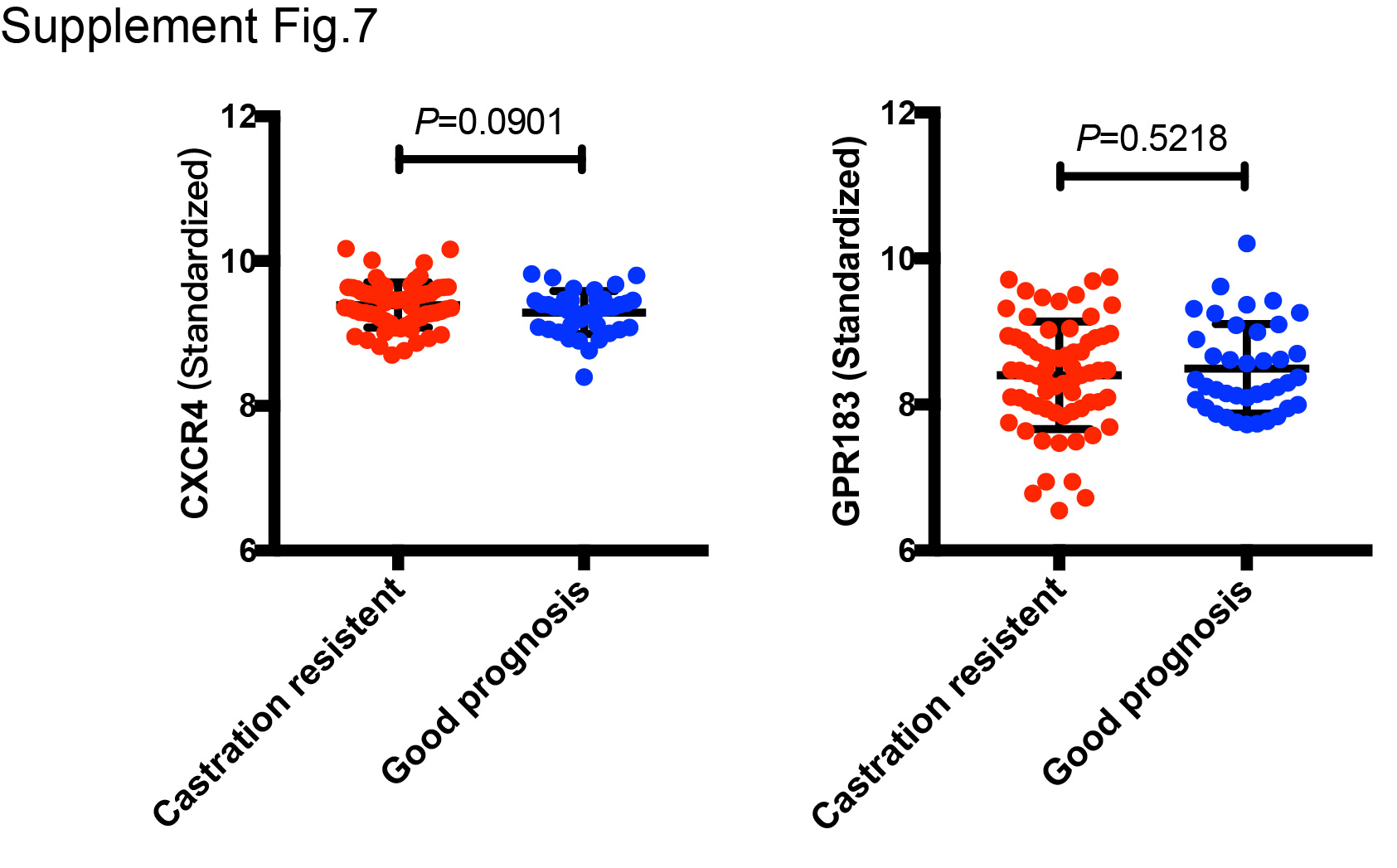

Supplement: Supplementary Figure 7 — Expression level of CXCR4 and GPR183 in blood between castration resistant patients and non-resistent patients. This data is re-analysied by us according to the GEO dataset GSE37199. [file Image_7.jpg]

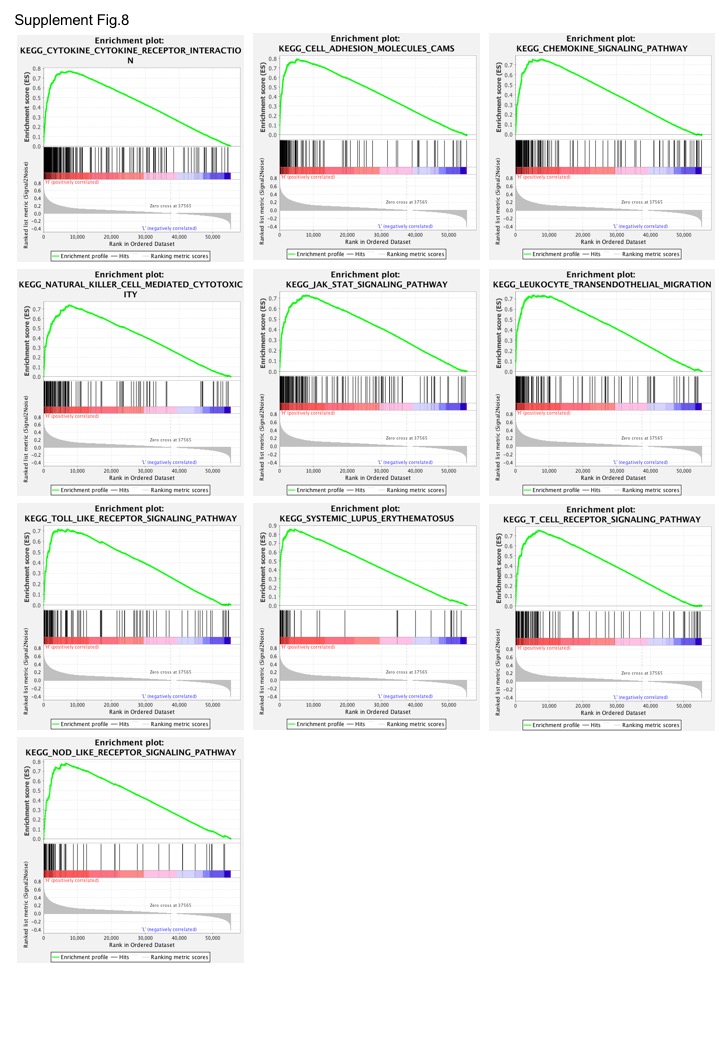

Supplement: Supplementary Figure 8 — Gene set enrichment analysis (GSEA) for the CXCR4 high-expressed group of PCa. Top10 enriched KEGG pathways are shown in this figure. [file Image_8.jpg]

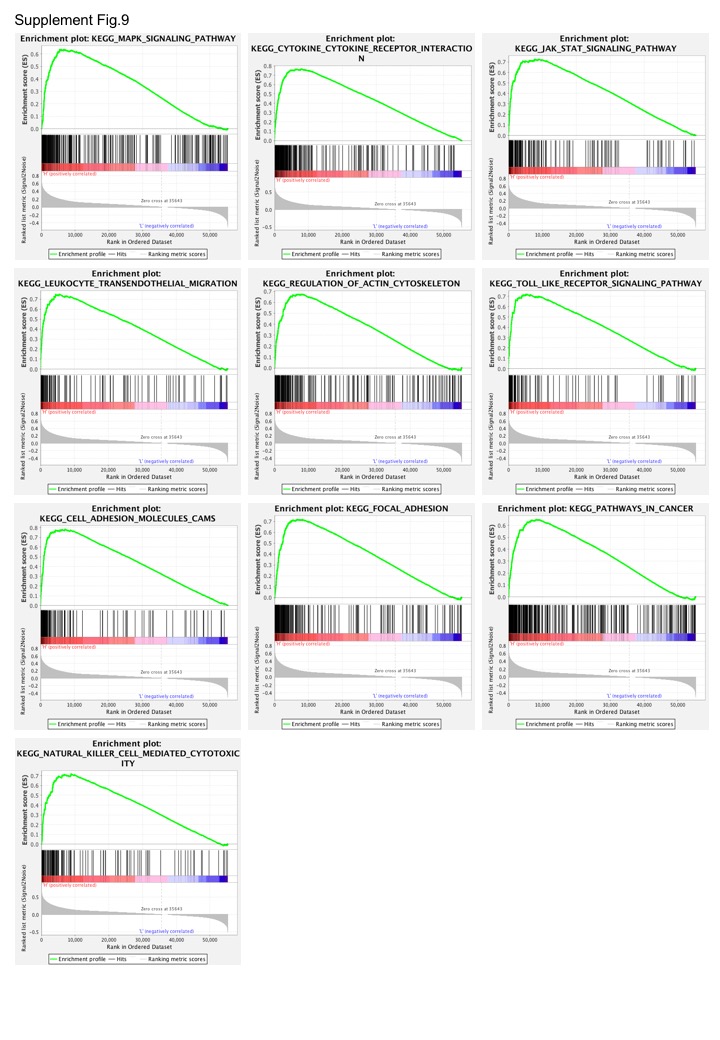

Supplement: Supplementary Figure 9 — Gene set enrichment analysis (GSEA) for the GPR183 high-expressed group of PCa. Top10 enriched KEGG pathways are shown in this figure. [file Image_9.jpg]
